# Different Forms of Ghrelin Exhibit Distinct Biological Roles in Tilapia

**DOI:** 10.3389/fendo.2013.00118

**Published:** 2013-09-03

**Authors:** Larry G. Riley

**Affiliations:** ^1^Department of Biology, California State University Fresno, Fresno, CA, USA

**Keywords:** ghrelin, GHS-R, tilapia, metabolism, appetite, homeostasis

## Abstract

Ghrelin has been identified in all vertebrate classes, including sharks. Each species possesses multiple forms of ghrelin that vary in peptide length and acyl modifications (e.g., *n*-hexanoic, *n*-non-anoic, *n*-octanoic, and *n*-decanoic acids) including des-acyl ghrelin. Octanoylated ghrelin has been shown to be a potent GH secretagogue, orexigenic factor, and plays a role in overall metabolism in vertebrates. In the tilapia model, octanoylated ghrelin (ghrelin-C8) and decanoylated ghrelin (ghrelin-C10) exhibit different biological actions. This mini review highlights the current knowledge of the differential actions of ghrelin-C8 and ghrelin-C10 from studies in the tilapia model. These findings suggest that the multiple forms of ghrelin may exhibit distinct yet complimentary actions directed toward maintaining overall energy balance in other vertebrates.

## Introduction

Ghrelin was first identified in rat stomach as an endogenous ligand for the growth hormone secretagogue receptor (GHS-R) (1). Ghrelin has since been identified in all vertebrate classes; fish (2–4), birds (5), amphibians (6), reptiles (7), mammals (1, 8), as well as sharks (9). All ghrelins identified thus far are uniquely and primarily acylated by octanoic or decanoic acid on the third amino acid from the N-terminus (10, 11). However, a variety of other acyl-forms of ghrelins (*n*-hexanoic and *n*-non-anoic acid) and unsaturated *n*-octanoic and *n*-decanoic isoforms of ghrelin have been identified (5, 12, 13). The acyl modification is necessary for ghrelins biological action (10). Indeed, the first seven amino acid residues on the N-terminus are highly conserved across vertebrates and are known as the “active core” (11, 14) suggesting an evolutionary conserved physiological role of ghrelin. Unlike tetrapod ghrelins, fish ghrelins possess an amide modification on the C-terminus (11). Ghrelin is predominately synthesized in the stomach and is also expressed in a variety of other tissues such as small and large intestine, pancreas, liver, hypothalamus, telencephalon, pituitary, gonads, kidneys, gills, adipose tissue, and many others (14, 15).

The biological actions of ghrelin are mediated by the GHS-R, which codes for two separate transcripts, GHS-R1a and GHS-R1b (16). GHS-R1a is a seven transmembrane domain G-protein coupled receptor. This receptor is responsive to both synthetic growth hormone secretagogues and ghrelin in regulating several neuroendocrine, metabolic, and non-endocrine actions (15). The GHS-R1b transcript is shorter than the GHS-R1a isoform due to the intron not being spliced out thus disrupting the normal reading frame and resulting in a “non-functional” receptor with five transmembrane domains (11, 17). GHS-R1b has been suggested to act as a dominant-negative mutant. The formation of GHS-R1a/GHS-R1b heterodimer facilitates the translocation of GHS-R1a to the nucleus decreasing the constitutive signaling of GHS-R1a, thus inhibiting ghrelin’s actions (18). Both isoforms are found in a variety of endocrine and non-endocrine tissues such as hypothalamus and a variety of other brain regions, pituitary, liver, lung, heart, muscle, kidney, and gonads (19). Two GHS-R isoforms have been identified in the black seabream (20). We have recently identified two GHS-R isoforms in the tilapia and determined their tissue distribution (21, 22).

The existence of ghrelin, GHS-R1a and GHS-R1b in fish suggests that the fundamental biological functions of ghrelin are conserved across vertebrate species (19, 20). In spite of the fact that all vertebrates possess multiple forms of ghrelin, nearly all of our understanding about ghrelin’s biological actions has come from studies using the ghrelin-C8, thus leaving a huge gap in our understanding of ghrelin biology. This mini review focuses on the differential effects of ghrelin-C8 and ghrelin-C10 in tilapia. For more general information on the structure and function of ghrelin within vertebrates the reader is referred to the following review papers (11, 14, 23, 24).

## Differential Roles of Ghrelin-C8 and Ghrelin-C10 in Tilapia

We have identified two forms of ghrelin in the Mozambique tilapia (*Oreochromis mossambicus*) stomach (4). They exhibit 100% amino acid identity with each other, the difference being the acyl modification (*n*-octanoic or *n*-decanoic) on Ser^3^. The major form of tilapia ghrelin possesses an *n*-decanoic (ghrelin-C10) modification (4). Multiple isoforms of ghrelin have been identified in other fish species, as observed in other vertebrates. Four isoforms of ghrelin have been identified in rainbow trout (2) and 11 isoforms of ghrelin have been identified in goldfish (25). In the chicken (5), ghrelin-C8 and ghrelin-C10 were isolated in similar amounts, whereas in goldfish (25), eel (3), bullfrog (26), and humans (27) ghrelin-C8 is the major form. Both acylated modifications are essential for receptor binding (28) and ghrelin transport across the blood-brain barrier (29).

Since its original discovery as a potent growth hormone secretagogue, ghrelin has been shown to be involved in a variety of neuroendocrine, metabolic, and non-endocrine functions that include, but not limited to, orexigenic activity, cardiovascular, gastrointestinal, pancreatic, and lipogenic and glucogenic actions (15, 16, 23, 30). In spite of the fact that all vertebrates studied to date possess multiple forms of ghrelin, nearly all of the published work has focused on the biological functions of ghrelin-C8 and des-acyl ghrelin. Hosoda et al. (27) have demonstrated that ghrelin-C8 and ghrelin-C10 exhibit the same potency to increase (Ca^2+^) levels in CHO cells expressing rat GHS-R1a as well as stimulate GH release in rats. In goldfish des-acyl ghrelin was shown to attenuate the orexigenic actions of ghrelin-C8, but had no effect on food intake when administered alone (31). Notwithstanding, since des-acyl ghrelin has been shown to exhibit some biological functions [e.g., stimulate adipogenesis and cardioprotective actions (32, 33)], that the biological role − beyond stimulating GH release − of the other ghrelin forms have not been further investigated.

We have shown in tilapia that ghrelin-C8 and ghrelin-C10 appear to exhibit differential biological functions (30). Ghrelin-C10 was more potent than ghrelin-C8 in stimulating GH release from the tilapia pituitary, yet neither form altered pituitary GH mRNA expression levels (21). Both forms equally increased liver IGF-1 mRNA expression, but ghrelin-C8 was more potent than ghrelin-C10 in increasing liver growth hormone receptor mRNA expression in tilapia (21). Tilapia treated with ghrelin-C10 for 21 days (via osmotic pumps) exhibited a significant increase in food intake and body weight; ghrelin-C8 had no effect. The increase in body weight was likely a result of increased adiposity in liver and muscle tissue induced by ghrelin-C10 (34). Ghrelin-C8 has been shown to stimulate adiposity in rat bone marrow (33).

Brain neuropeptide Y (NPY) mRNA expression levels were significantly elevated 4 and 8 h following ghrelin-C10, not ghrelin-C8, injection in tilapia (30). In goldfish, the orexigenic actions of ghrelin have been shown to be mediated by the NPY pathway (35), thus suggesting a similar mechanism of control in tilapia. In spite of the orexigenic actions of NPY in vertebrates (36), we have not observed an acute increase in food intake following either ghrelin-C8 or ghrelin-C10 treatment (unpublished observations). In rainbow trout, ghrelin-C8 treatment has been shown to have no effect on food intake (37), increase food intake (38), and inhibit food intake (37). In goldfish, two forms of octanoylated ghrelin (12- and 17-amino acid residues) stimulated food intake, whereas des-acylated ghrelin17 had no effect (25). Ghrelin-C8 treatment has routinely been shown to stimulate food intake in mammals (24, 39). However, studies using ghrelin (*ghrl*^−/−^) knockout models (40, 41) suggest that ghrelin’s role in stimulating food intake is secondary to its maintenance of metabolic energy balance (16, 42). The use of *ghrl*^−/−^ models provides a unique opportunity to investigate the differential roles of the multiple forms of ghrelin.

**Figure 1:**
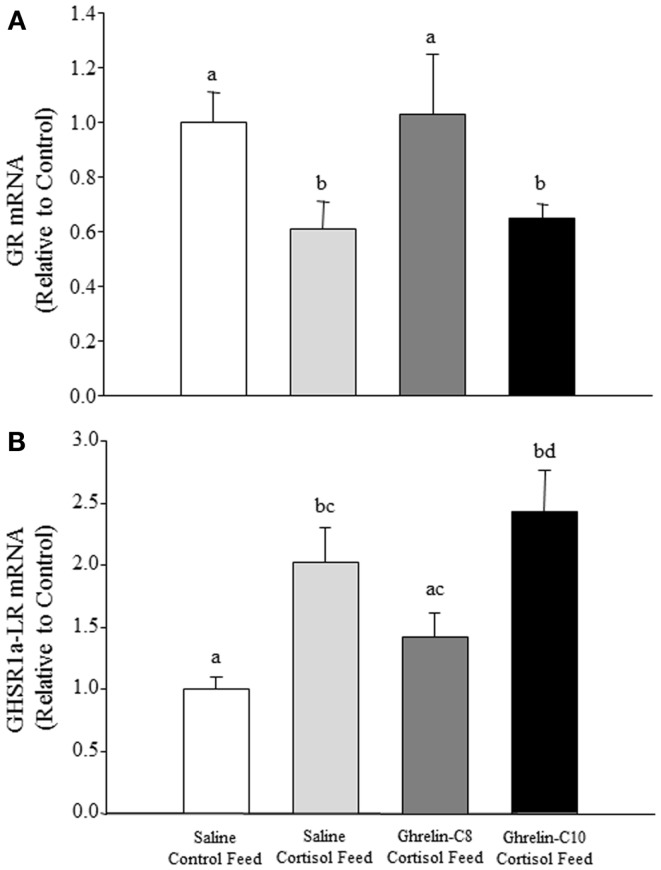
**Sexually mature male and female tilapia were surgically implanted with a micro-osmotic pump into the IP cavity containing either saline (control), 100 μg/ml of ghrelin-C8, or 100 μg/ml of ghrelin-C10 (34)**. The calculated rate of release at 24°C was 13 ng/h for 32 days. Twenty-four hours following the surgery fish were fed a control diet or cortisol-laden feed [500 mg/kg feed; (48)] for 21 days twice a day. Upon termination of the experiment brain sections were collected, RNA was extracted and reversed transcribed into cDNA. Relative mRNA expression levels were determined by qPCR. Ghrelin-C8 treatment significantly reversed the inhibitory effects of cortisol on GR mRNA expression levels in the hypothalamus **(A)**. Ghrelin-C8 treatment partially reversed the stimulatory effect cortisol exhibited on hypothalamic GHS-R-1a-LR mRNA expression levels **(B)**. mRNA data are presented as relative to saline control feed group. Columns with different letters are significantly different at *P* < 0.05, *n* = 10–12.

In tilapia, only ghrelin-C8 significantly elevated plasma glucose levels 4 and 8 h post intraperitoneal injection (30). In rainbow trout ghrelin-C8 stimulated glucokinase (GK) and pyruvate kinase activity, as well as increased the mRNA expression levels of glucose transporter-2 and *GK* in different regions of the brain, without altering plasma glucose levels (43). These data suggest that ghrelin-C8, in tilapia and rainbow trout, may play a role in central glucose-sensing as well as in glucose metabolism in fish as observed in mammals (44). Des-acyl ghrelin had no effect on plasma glucose or insulin levels in healthy humans, but counteracted the actions of ghrelin-C8 on glucose and insulin levels (45). This indicates that des-acyl ghrelin possesses metabolic functions in mammals (33, 45). These data lend support to the hypothesis that the other acyl-forms of ghrelin may exhibit distinct functions from that of *n*-octanoylated ghrelin. Recently, we have observed that ghrelin-C8 reversed the negative effects of cortisol on the mRNA expression levels of the glucocorticoid receptor (GR) and GHS-R1a-LR in the hypothalamus of tilapia (Figures 1A,B, respectively). These data suggest that ghrelin-C8 may be playing a role in counteracting the negative effects of chronic stress and/or stress recovery in tilapia. We have previously observed differential regulation of the GHS-R mRNA isoforms in tilapia (46, 47). Further studies are needed to elucidate the biological significance of the different expression patterns of the GHS-Rs. Taken together, our data in tilapia clearly shows that ghrelin-C8 and ghrelin-C10 exhibit distinct, yet complimentary actions directed toward maintaining metabolic balance within the animal. Our laboratory is currently investigating the direct effects of ghrelin-C8 and ghrelin-C10 on neuropeptide mRNA expression patterns using brain tissue culture methods and proteomic and metabolic approaches.

## Conclusion

To date, all vertebrates produce multiple forms of ghrelin. There are reports that des-acyl ghrelin exhibits biological functions in mammals (32, 33) and that both ghrelin-C8 and ghrelin-C10 simulate GH release in rats (27), suggesting that the other acyl-forms of ghrelin likely exhibit biological functions. In tilapia, both ghrelin-C8 and ghrelin-C10 exert distinct biological actions that appear to be directed toward maintenance of metabolic balance. It is not clear what is the mechanism underlying the different biological effects of ghrelin-C8 and ghrelin-C10. A possible hypothesis is that a third GHS-R-isoform that exhibits higher affinity toward ghrelin-C10 exists or that ghrelin-C10 binds non-specifically to a related receptor.

## Conflict of Interest Statement

The author declares that the research was conducted in the absence of any commercial or financial relationships that could be construed as a potential conflict of interest.
